# Theoretical Study on the Gas-Phase and Aqueous Interface Reaction Mechanism of Criegee Intermediates with 2-Methylglyceric Acid and the Nucleation of Products

**DOI:** 10.3390/ijms24065400

**Published:** 2023-03-11

**Authors:** Lei Li, Qingzhu Zhang, Yuanyuan Wei, Qiao Wang, Wenxing Wang

**Affiliations:** Environment Research Institute, Shandong University, Qingdao 266237, China; lilei0928@mail.sdu.edu.cn (L.L.); m15621010095@163.com (Y.W.); wangqiao@mee.gov.cn (Q.W.); wxwang@sdu.edu.cn (W.W.)

**Keywords:** Criegee intermediates, 2-methylglyceric acid, gas-phase reaction, functional group effect, gas-liquid interface reaction

## Abstract

Criegee intermediates (CIs) are important in the sink of many atmospheric substances, including alcohols, organic acids, amines, etc. In this work, the density functional theory (DFT) method was used to calculate the energy barriers for the reactions of CH_3_CHOO with 2-methyl glyceric acid (MGA) and to evaluate the interaction of the three functional groups of MGA. The results show that the reactions involving the COOH group of MGA are negligibly affected, and that hydrogen bonding can affect the reactions involving α-OH and β-OH groups. The water molecule has a negative effect on the reactions of the COOH group. It decreases the energy barriers of reactions involving the α-OH and β-OH groups as a catalyst. The Born-Oppenheimer molecular dynamic (BOMD) was applied to simulate the reactions of CH_3_CHOO with MGA at the gas-liquid interface. Water molecule plays the role of proton transfer in the reaction. Gas-phase calculations and gas-liquid interface simulations demonstrate that the reaction of CH_3_CHOO with the COOH group is the main pathway in the atmosphere. The molecular dynamic (MD) simulations suggest that the reaction products can form clusters in the atmosphere to participate in the formation of particles.

## 1. Introduction

Plants can produce large amounts of volatile organic compounds (VOCs), and alkenes are an important component of VOCs [1]. The ozonolysis of alkenes is the main source of Criegee intermediates (CIs) in the atmosphere [2]. The first step of this process is the opening of the double bond of the alkene, where the oxygens at the ends of the ozone molecule combine with the carbons on either side of the double bond to form the primary ozonide (POZ), which decomposes into a carbonyl compound and an excited CI [3]. The excited CIs can be decomposed into OH radicals [4]. The unimolecular reaction of CIs is an important pathway for generating OH radicals in the atmosphere. Although CI decomposition contributes only about 24% of the OH production during the daytime, it is responsible for most OH formation at night [5]. Atmospheric tropospheric organic chemistry experiments conducted near London, UK, in 2003 showed that alkene ozonolysis accounted for about one-third of the atmospheric source of OH radicals during the day, and at night the average concentration of OH radical was about 2.6 × 10^5^ molecule cm^−3^, with almost all OH radicals coming from alkene ozonolysis [6,7]. In the Chilean observations, olefin ozonolysis contributed about a quarter of the OH radicals in the atmosphere, with internal alkenes contributing 86%. The OH radical yield of internal alkenes was much higher than that of terminal alkenes [8]. The box modeling showed that the decomposition of CI is the main source of OH in the summer and winter PUMA (Pollution of the Urban Midlands Atmosphere) campaign [9]. Therefore, the decomposition of CI has a profound effect on the oxidation capacity of the troposphere.

However, partially excited CIs lose energy through collisional quenching to produce stabilized CIs [10]. Stabilized CIs have the opportunity to participate in bimolecular reactions in the atmosphere, including the reactions with H_2_O, NO_x_, SO_2_, alcohols and carboxylic acids etc. [11,12,13,14]. The abundance of water vapor is high in the troposphere, so water is the most critical scavenger for CIs [15]. The primary product of the reaction is hydroxymethyl hydroperoxide (HMMP), which has toxic effects on plants [16,17]. HMHP can decompose to form OH radicals or decompose to water molecules and formic acid. GEOS-Chem model simulations and theoretical calculations prove that this process is one of the sources of formic acid in the atmosphere, and HMMP contributes to the formation of secondary organic aerosol (SOA) [18,19]. The reaction products of some specially structured CIs with water are important to cloud condensation nuclei in the atmosphere [20]. The CI-NO_2_ reaction produces NO_3_, which affects the oxidation capacity of the atmosphere at night [21]. CIs oxidize SO_2_ to produce sulfuric acid and sulfate, which are important contributors to the formation of SOA [22,23,24]. The reaction with alcohol is one of the pathways for the sink of atmospheric CIs. The reaction products of CIs with alcohols are alpha-alkoxyalkyl hydroperoxides (AAAHs), and the oxidation products of AAAHs have an important influence on the formation of SOA in the atmosphere [3,25]. The reactions between CIs and alcohols are sensitive to humidity, and even a single water molecule can greatly facilitate the reaction by forming a ring structure. The reaction between *syn*-CH_3_CHOO and CH_3_OH is three times faster when a single water molecule is involved as a catalyst [26]. It greatly enhances the contribution of the reaction with alcohols for the removal of CIs. The α-acyloxyalkyl-1-hydroperoxides, formed by the reaction of CIs with organic acids, have low volatility and can promote the formation of organic aerosols [27,28].

In addition to bimolecular reactions, some studies focused on the effect of different structures of CIs on the reactions. For example, Yin and Takahashi et al. [29] investigated the reactions of different substituent CIs with water. The results showed that saturated CIs were more reactive than unsaturated species. The behavior of CIs with different conformations was studied and compared with other methyl-substituted CIs [30]. However, the studies on the functional group effects of reactants in CI reactions are limited. 2-Methylglyceric acid (MGA) is a kind of highly oxygenated organic molecule (HOMs) in the atmosphere [31]. MGA is derived from the oxidation of isoprene and has been repeatedly detected in smog chambers, terrestrial and even marine atmospheric SOA [32,33,34,35,36]. It is an important oxidation product of isoprene and a tracer of isoprene-derived SOA [33,37]. MGA is often found in atmospheric observations and laboratory studies of aerosol formation cases, indicating that it is a significant potential substance involved in SOA formation [32,38]. In the 2003 cruise observation from the Bohai Sea to the Arctic, MGA accounted for a major proportion of the SOA tracers [35]. MGA also made a significant contribution to the SOA samples collected from the marine boundary layer from the Arctic to the Antarctic [36]. It has three functional groups: two hydroxyl groups are located on different carbons, and one carboxyl group resides on one side of the molecule. Here, we investigated the reactions of CIs with MGA and the effect of functional groups on the reaction energy barriers. And the reaction products of MGA may also form hydrogen bonds with other nucleating molecules due to multiple functional groups.

In this work, C2 CIs were selected as reactants, which were shown to have a longer lifetime than CH_2_OO at the gas-liquid interface. The density functional theory (DFT) was used to explore the reaction energy barriers and interactions of the individual functional groups of MGA. Meanwhile, the role of water molecules in the gas phase reactions was investigated. The ab initio dynamic was used to simulate the gas-liquid interface reactions of CH_3_CHOO with MGA. Finally, the nucleation process of the reaction products was performed by molecular dynamics (MD) to examine the ability to form particles.

## 2. Results and Discussion

**Figure 1:**
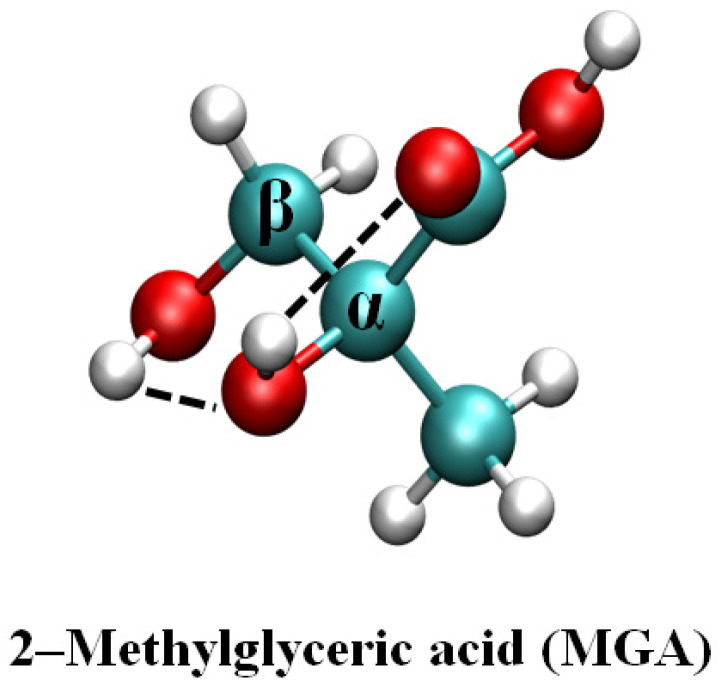
The most stable structure of MGA.

The lowest energy configuration of MGA is shown in Figure 1. The hydrogen of the beta-hydroxyl group (β-OH) forms a hydrogen bond (2.26 Å) with the oxygen of the alpha-hydroxyl group (α-OH), and the hydrogen on the α-OH forms a hydrogen bond (2.08 Å) with the carbonyl oxygen of the carboxyl group. The hydrogen bonds contribute to the stability of the MGA molecule.

### 2.1. Gas-Phase Reactions

#### 2.1.1. Bimolecular Reactions

**Figure 2:**
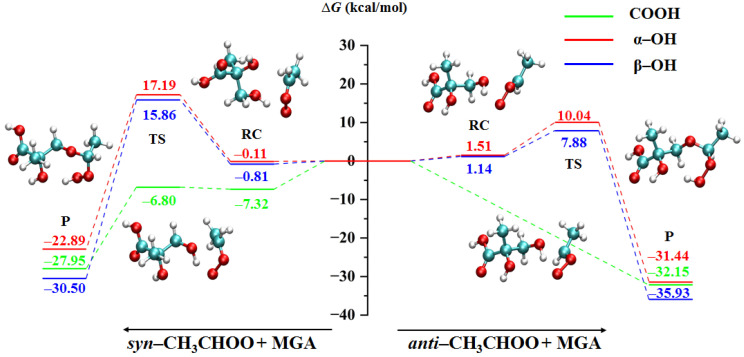
The minimum energy pathway for the CH_3_CHOO + MGA reactions (RC: reaction complex, TS: transition state, P: product).

In the reactions of *syn*- and *anti*-CH_3_CHOO with the three functional groups of MGA, the reaction transition state of *anti*-CH_3_CHOO with the COOH group was not observed. As shown in Figure 2, the energy of the reaction complexes is considered a benchmark. The reaction of *anti*-CH_3_CHOO with the COOH group is barrierless. The reaction of *syn*-CH_3_CHOO with the COOH group is almost barrierless (0.52 kcal/mol). This result is consistent with previous studies that the reactions of C1 and C2 CIs with formic and acetic acids are barrierless pathways [39,40]. The reaction energy barriers of *syn*-CH_3_CHOO with α-OH and β-OH groups are 17.30 and 16.67 kcal/mol, respectively. Similarly, the reaction energy barrier of *anti*-CH_3_CHOO + α-OH (8.53 kcal/mol) is slightly larger than that of *anti*-CH_3_CHOO + β-OH (6.74 kcal/mol). This suggests that the β-OH group is more energetically favorable to react with CH_3_CHOO than the α-OH group. In the reactions of COOH, α-OH and β-OH groups of MGA, the energy barriers of *anti*-CH_3_CHOO (0.00, 8.53, 6.74 kcal/mol) are lower than that of *syn*-CH_3_CHOO (0.52, 17.30, 16.67 kcal/mol), indicating that the reactivity of *anti*-CH_3_CHOO is higher than that of *syn*-CH_3_CHOO. The COOH group of MGA prefers to react with CH_3_CHOO compared with the other two functional groups in the atmosphere. The structures of all reaction complexes, transition states and products are displayed in Figure S2.

To explore the effect of functional groups on the reaction energy barrier, one or two functional groups in MGA are replaced by H atoms. Then the energy barriers for the reactions of CH_3_CHOO with these reactants were calculated. The structures of these reactants and reaction energy barriers are given in Table 1. In all the reactions of the COOH group, the reaction energy barriers are zero or nearly zero kcal/mol, which indicates that α-OH and β-OH groups do not affect the reaction energy barriers of the COOH group and the reactions of CIs with the COOH group are very active. For the reactions of the α-OH group, the energy barrier of the *syn*-CH_3_CHOO reaction decreases by 0.47 kcal/mol as the β-OH group of MGA is replaced by an H atom. And the energy barriers of the R4 and R5 reactions decrease by 3.63 and 3.93 kcal/mol as the COOH group of MGA is replaced by the H atom. For *anti*-CH_3_CHOO reactions, the energy barrier lowered in the R2 reaction (0.07 kcal/mol) is also smaller than that of the R4 and R5 reactions (0.69 and 0.54 kcal/mol), indicating that the COOH group inhibited the reactions of the α-OH group more strongly than the β-OH group. The loss of hydrogen bond between the α-OH and the COOH group makes it easier for the H of the α-OH group to transfer to the terminal oxygen of CH_3_CHOO. For the reactions of *syn*-CH_3_CHOO with the β-OH group, due to the long distance of the COOH group from the β-OH group, the reaction energy barrier is reduced by 1.37 kcal/mol as the H atom replaces the COOH group. The reaction energy barriers are reduced by 3.00 and 4.02 kcal/mol (R1 and R6 reactions) as the α-OH group is replaced by an H atom. The hydrogen bond between the α-OH and β-OH group also increases the reaction energy barriers of the β-OH group. However, in the reaction of *anti*-CH_3_CHOO, the energy barriers of the R1 and R4 reactions are increased. This may be due to the increased distance between the H of the β-OH group and the O of the α-OH group (2.46 Å) when *anti*-CH_3_CHOO forms a complex with MGA (Figure S2), and it is easier for the addition of the β-OH to *anti*-CH_3_CHOO.

#### 2.1.2. Water-Mediated Reactions

**Figure 3:**
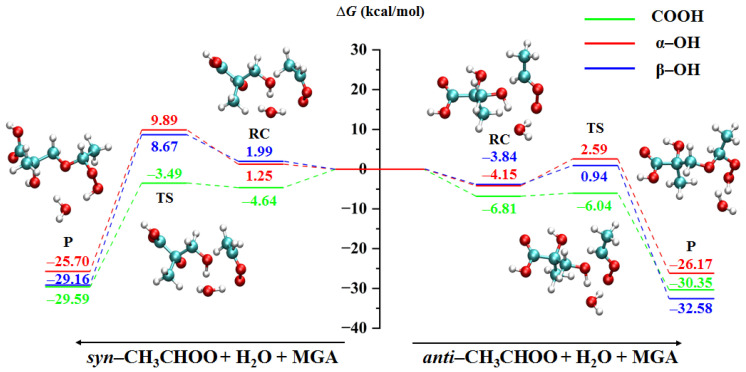
The minimum energy pathway for the CH_3_CHOO + H_2_O + MGA reactions (RC: reaction complex, TS: transition state, P: product).

The minimum energy pathway for the water-mediated reactions of CH_3_CHOO with MGA is presented in Figure 3. For the reactions involving the COOH group, the participation of the water molecule increases the reaction energy barriers. The energy barriers of reactions involving *syn*- and *anti*-CH_3_CHOO are increased by 0.63 and 0.77 kcal/mol, respectively. In contrast, the water molecule decreases the energy barriers of the reactions of α-OH and β-OH groups, which acts as a catalyst. The reaction energy barriers are decreased by 8.66 and 9.99 kcal/mol for the cases of *syn*-CH_3_CHOO with α-OH and β-OH groups, respectively. The energy barriers of *anti*-CH_3_CHOO interacting with α-OH and β-OH groups are decreased by 1.79 and 1.96 kcal/mol, respectively. The promoting effect of the water molecule for reactions of *syn*-CH_3_CHOO is stronger than that for *anti*-CH_3_CHOO. The water-mediated reactions also suggested that the reactivity of *anti*-CH_3_CHOO is greater than that of *syn*-CH_3_CHOO. The structures of all water-mediated reaction complexes, transition states and products are shown in Figure S3.

### 2.2. Gas-Liquid Interface Reactions

The gas-liquid interface significantly impacts the chemical reactions in the atmosphere [41,42,43]. In this study, CH_3_CHOO and MGA molecules were placed on a droplet composed of thirty water molecules to explore the effect of the gas-liquid interface on the reactions. To eliminate the impact of droplet position on the reactions, the simulations of reactions involving *syn*- and *anti*-CH_3_CHOO were performed thirty times, each at different positions.

#### 2.2.1. Reaction of Anti-CH_3_CHOO with the MGA COOH Group

**Figure 4:**
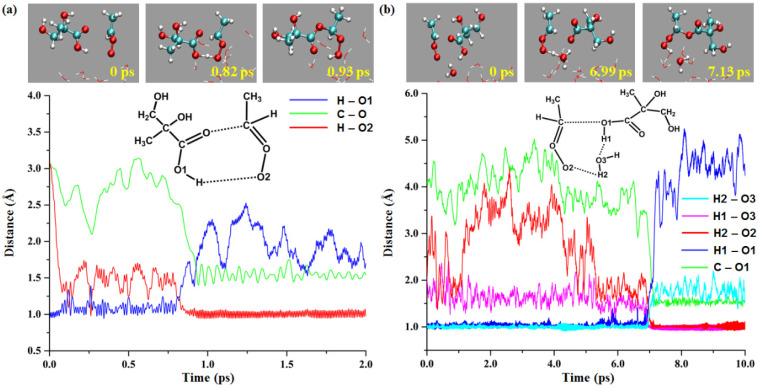
The direct and water-mediated reaction processes of *anti*-CH_3_CHOO with MGA COOH group at the gas-liquid interface (**a**): direct reaction, (**b**): water-mediated reaction.

Figure 4 shows the key bond length variation and snapshots of the reaction between the *anti*-CH_3_CHOO and COOH group of MGA at the gas-liquid interface. As presented in Figure 4a, the distances of H-O1, C-O and H-O2 are 0.97, 3.07 and 3.07 Å, respectively, at 0 ps. Subsequently, the H of the COOH group gradually approaches the terminal oxygen of *anti*-CH_3_CHOO and the distance of C-O decreases at 0.55 ps. At 0.82 ps, a transition-state-like structure is formed between the two molecules. The distances of H-O1, C-O and H-O2 are 1.20, 2.50 and 1.23 Å, respectively. At 0.93 ps, the C-O and O-H2 bonds are formed and stabilized, indicating the formation of the product. The water-mediated reaction process is shown in Figure 4b. At 0 ps, the distances of H2-O3, H1-O3, H2-O2, H1-O1 and C-O1 are 1.00, 2.08, 2.29, 0.97 and 4.12 Å, respectively. The transition-state-like structure appears at 6.99 ps. The water molecule is located between H1 and O2 to transfer the proton. And the distances of H2-O3, H1-O3, H2-O2, H1-O1 and C-O1 are 1.22, 1.03, 1.22, 1.74 and 2.68 Å, respectively. At 7.13 ps, the proton transfer is completed, and the additional product is generated. The distances of H2-O3, H1-O3, H2-O2, H1-O1 and C-O1 are 1.49, 1.02, 1.01, 2.48 and 1.56 Å, respectively. The water molecule becomes the bridge for proton transfer during the reaction. The direct reaction of the COOH group was completed within 1 ps, and the water-mediated reaction was completed at about 7 ps, with a time difference of about 6 ps between the two reactions. In previous studies, the direct reactions of CH_2_OO and CH_3_CHOO with formic and acetic acids were extremely fast, with rate constants greater than 1 × 10^−10^ cm^3^ molecule^−1^ s^−1^ and reaction rates close to the collision limit [40]. Moreover, the reaction of CH_2_OO with formic acid has been shown to be a barrierless process [44]. In the present study, the direct reaction of the COOH group in the gas phase is also barrierless, and the energy barrier rises after adding water molecules, which are unfavorable to the reaction. Based on the above description, it is highly likely that the direct reaction of the COOH group is faster than the water-mediated reaction. The key bond length variations and snapshots of the gas-liquid interfacial reaction of *syn*-CH_3_CHOO with the COOH group of MGA are depicted in Figure S4. The reactions of CH_3_CHOO with the α-OH and β-OH groups of MGA and the water-mediated reaction of *syn*-CH_3_CHOO with the COOH group of MGA were not observed in the simulations.

The gas-phase reaction and the BOMD simulation results are in good agreement, with the reactions of the COOH group, which has a lower energy barrier, occurring more easily in the BOMD simulations. On the other hand, the reactions of the OH group have high energy barriers that are difficult to observe in the simulations.

#### 2.2.2. MGA-Mediated Hydration of Anti-CH_3_CHOO

**Figure 5:**
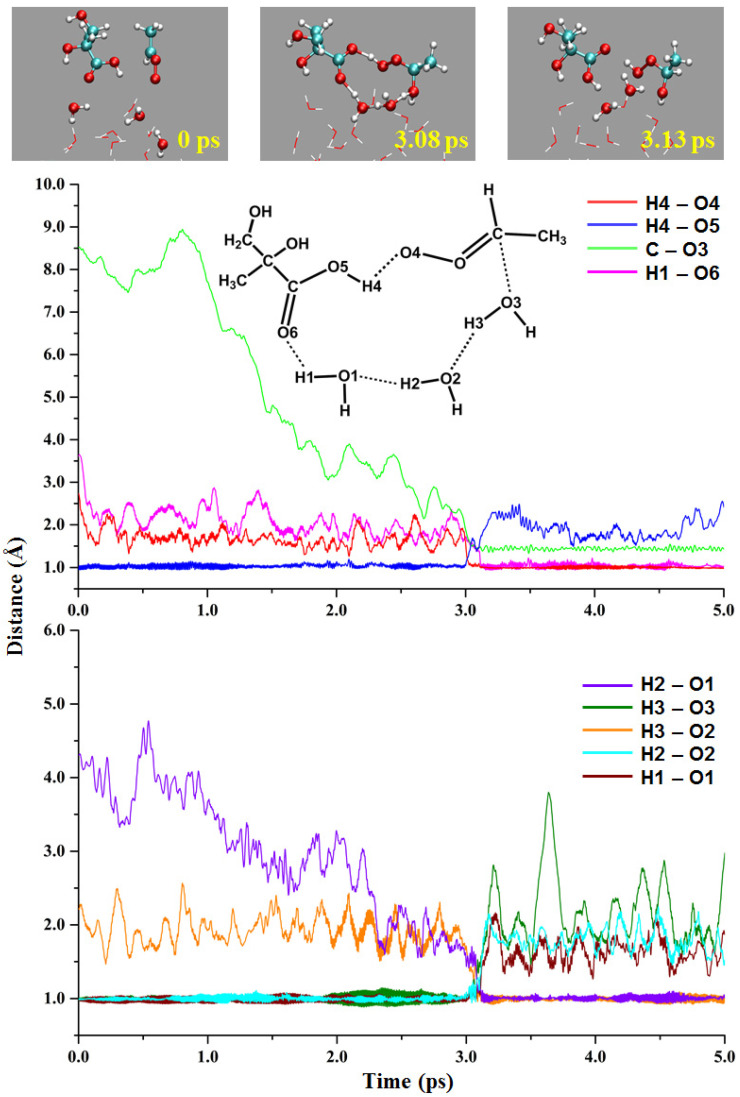
The MGA-mediated hydration of *anti*-CH_3_CHOO at the gas-liquid interface.

The reaction of CIs with water is the most important sink pathway in the atmosphere [45,46,47]. In the simulations, MGA-mediated hydration of *anti*-CH_3_CHOO as a catalyst was observed. As shown in Figure 5, the initial distances of the H4-O4, H4-O5, C-O3 and H1-O6 are 2.70, 0.97, 8.52 and 3.61 Å, respectively. The transition-state-like structure appears at 3.08 ps, where *anti*-CH_3_CHOO, MGA and three water molecules form a ring structure. The H4-O4, H4-O5, C-O3 and H1-O6 distances are 1.03, 1.44, 1.46 and 1.41 Å, respectively. The proton transfer of the three water molecules and the COOH group occur almost simultaneously. At 3.13 ps, the distances of H4-O4, C-O3 and H1-O6 are stable at 1.00, 1.38 and 1.00 Å, indicating the formation of the product.

### 2.3. Nucleation of Products

**Figure 6:**
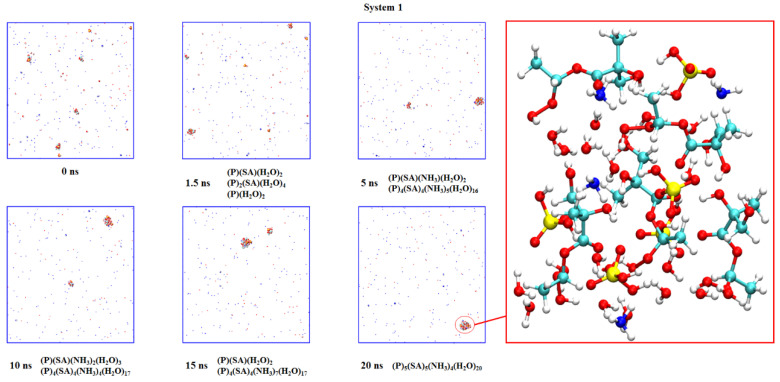
The snapshots of nucleation simulation for the reaction product of *anti*-CH_3_CHOO + MGA-COOH.

It is reported that the CI reactions are likely to generate low volatile products, which can participate in the nucleation process and form SOA in the atmosphere. The reaction product of CIs and peroxyl radicals is an oligomer, one of the chemical compositions of SOA [48]. In the gas phase, the continuous insertion of CIs into the hydroperoxide esters leads to the formation of oligomers that make an important contribution to the generation of SOA [49]. This study simulated the nucleations of CH_3_CHOO + MGA reaction products (P) with NH_3_, sulfuric acids (SA) and H_2_O. Initially, five products, ten NH_3_, five SA, twenty H_2_O, one hundred and fifty-four N_2_ and forty-one O_2,_ were randomly put into the supercell. The nucleation simulation for the reaction product of *anti*-CH_3_CHOO + MGA-COOH is shown in Figure 6. At 1.5 ns, there are three clusters in the system, which are (P)(SA)(H_2_O)_2_, (P)_2_(SA)(H_2_O)_4_ and (P)(H_2_O)_2_. The NH_3_ is not adsorbed into the clusters. The three clusters grow into two larger clusters at 5 ns. Afterwards, there are always two clusters in the system whose composition changes continuously. However, the number of P and SA remained constant, indicating that P binds more strongly to SA than NH_3_ and H_2_O during nucleation. At 20 ns, the (P)_5_(SA)_5_(NH_3_)_4_(H_2_O)_20_ cluster is formed in the system. The nucleation simulations of the other five products are depicted in Figures S5–S9.

## 3. Methods and Materials

Twelve different structures of MGA molecules were optimized, and six different configurations of MGA molecules were obtained (Figure S1). The MGA molecule with the lowest energy was selected as the reactant (MGA-6). The structural optimization of the reactants, reaction complexes, transition states, and products and the transition state searching was conducted using the M06-2X functional [50] in combination with the 6-31+G(d,p) basis set. The intrinsic reaction coordinates (IRC) calculation was used to confirm that the transition states were connected to the correct reaction complexes and products [51,52]. The single-point energies of all structures were performed at the M06-2X/6-311++G(3df,3pd) level. Gaussian 09 software was used to calculate the electronic structures of all gas-phase reactions [53]. All gas-phase reaction calculations are based on the DFT method. DFT is based on the Hohenberg-Kohn theorem, which describes and determines a system’s ground state energy and other physical quantities by electron density and applies the Kohn-Sham equation to calculate real molecular systems. DFT is based on the Hohenberg-Kohn theorem, which describes and determines a system’s ground state energy and other physical quantities by electron density and applies the Kohn-Sham equation to calculate real molecular systems. DFT simplifies the Schördinger wave function solution process and solves the 3N-dimensional wave function problem with a three-dimensional electron density problem. The equation for the total energy is:

$$\begin{array}{l} E(\rho )=-\frac{\hbar^{2}}{2m_{e}}\sum_{i=1}^{n}\int \psi_{i}^{*}(r_{1})\nabla_{1}^{2}\psi_{i}(r_{i})dr_{i}-\sum_{I=1}^{N}\int \frac{Z_{I}e^{2}}{4\pi \varepsilon_{0}r_{I1}}\rho (r_{1})dr_{1}\\ +\frac{1}{2}\int \frac{\rho (r_{1})\rho (r_{2})e^{2}}{4\pi \varepsilon_{0}r_{12}}dr_{1}dr_{2}+E_{XC}[\rho ] \end{array}$$

The items on the right side of the equation are the electron kinetic energy of the system, the attraction potential between the nucleus and the electron, the Coulomb interaction between the charges and the exchange-interaction energy. The electron density equation is:

$$\rho (r)=\sum_{i=1}^{n}{\left[\psi_{i}(r)\right]}^{2}$$

All gas-liquid interface reactions were performed with Born-Oppenheimer molecular dynamics (BOMD) using CP2K software based on the DFT method [54]. The simulations were carried out in supercell with a side length of 35 Å and containing thirty water molecules. The electronic exchange interaction was treated by the Becke-Lee-Yang-Parr (BLYP) functional [55,56]. The valence electron was treated by a double-ζ Gaussian basis set (DZVP) [57]. The core electron was treated by Goedecker-Teter-Hutter (GTH) norm-conserved pseudopotentials [58,59]. The integration step is set to 1 fs. The simulations were all performed using a constant volume and temperature (NVT) ensemble. The Nose-Hoover chain method was used to maintain the system temperature at 298 K. The ab initio method used in the simulations performs the all-electron system Schördinger equation for the studied system to solve the energy and wave functions. The Schördinger equation was proposed by the Austrian physicist Schrödinger in 1926. The equation is:

$$i\hbar \frac{\partial }{\partial t}\psi (r,t)=\hat{H}\psi (r,t)$$

with

$$\hat{H}=-\frac{\hbar^{2}}{2m}\nabla^{2}+V(r,t)$$

The nucleation process was calculated using the GROMACS 5.1.0 [60] program. MD simulation was performed in a 200 × 200 × 200 Å^3^ supercell. The electrostatic potentials of all substances in the supercell were calculated at the M06-2X/6-31+G(d,p) level. The first step was energy minimization. Then, the NVT ensemble was carried out for 100 ps, and constant pressure and temperature (NPT) were carried out for 20 ns. Berendsen pressure coupling was employed on the pressure coupling [61]. The velocity rescaling thermostat was employed on the temperature coupling [62]. The step size of the dynamic’s simulation was set to 1 fs. Particle-mesh Ewald (PME) algorithm was performed to handle long-range electrostatic interactions [63]. The bonding length constraint was handled using the LINCS algorithm [64].

## 4. Conclusions

Based on reaction energy barriers calculated in the present work, the reactions with the MGA COOH group are the dominant pathways for CH_3_CHOO interactions in the gas phase. The energy barriers of the reaction involving the α-OH group are higher than that of the β-OH group due to the binding of the two hydrogen bonds. And the reactions of *anti*-CH_3_CHOO are more favorable than that of *syn*-CH_3_CHOO. For the effect of functional groups, the α-OH and β-OH groups have almost no impact on the reaction of the MGA COOH group, which is the most active functional group in the reactions with CH_3_COOH. Either the α-OH or β-OH group is affected by the other two functional groups. The presence of the other functional groups almost always inhibits the reactions of the α-OH or β-OH group. The water molecule inhibits the reactions of the MGA COOH group but promotes the reactions of the α-OH and β-OH groups. At the gas-liquid interface, the reactions of the COOH group are also the most dominant pathways, and the water molecule is the bridge for proton transfer. The MGA COOH group can play the role of water molecule involved in the hydration of CH_3_CHOO. Since the direct reaction of CIs with the MGA COOH group is the dominant pathway, dry atmospheric environments are more favorable for the reaction of CIs with MGA. In humid and CI-rich atmospheres (e.g., tropical rainforest regions), water-mediated reactions with MGA-COOH and MGA-mediated hydration are also sinks for CIs. The reaction products of CH_3_CHOO and MGA can form clusters with ammonia, sulfuric acids, and water molecules, which is a significant step in the formation of new particles in the atmosphere. Both the MGA and the reaction products contribute to SOA pollution.

## Figures and Tables

**Table 1:** The reaction energy barriers of CH_3_CHOO with the reactants obtained by replacing the functional groups of MGA with H atom.

Functional Groups	Reactant Serial Numbers	Reactants	Reaction Energy Barriers (kcal/mol)	
			*Syn*-CH_3_CHOO	*Anti*-CH_3_CHOO
COOH	R1	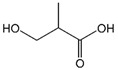	0.00	0.00
	R2	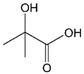	0.06	0.00
	R3	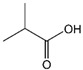	0.00	0.00
α-OH	R4	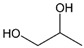	13.67	7.84
	R2	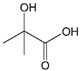	16.83	8.46
	R5	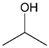	13.37	7.99
β-OH	R4	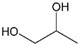	15.30	9.63
	R1	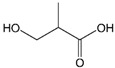	13.67	7.52
	R6	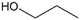	12.65	5.90

## Data Availability

Not applicable.

## Supplementary Materials

The following supporting information can be downloaded at: https://www.mdpi.com/article/10.3390/ijms24065400/s1.
