# Supplementary material for: Theoretical Study on the Gas-Phase and Aqueous Interface Reaction Mechanism of Criegee Intermediates with 2-Methylglyceric Acid and the Nucleation of Products
Source: Int J Mol Sci. 2023 Mar 11;24(6):5400. doi: 10.3390/ijms24065400 (PMC10049390; doi:10.3390/ijms24065400)
Supplement: Supplementary file 1 [file ijms-24-05400-s001.zip › ijms-2269151-supplementary.pdf]

Supplementary Material

Theoretical Study on the Gas-Phase and Aqueous Interface Reaction Mechanism of Criegee Intermediates with 2-Methylglyceric Acid and the Nucleation of Products

Lei Li, Qingzhu Zhang *, Yuanyuan Wei, Qiao Wang and Wenxing Wang

Environment Research Institute, Shandong University, Qingdao 266237, China

* Correspondence: zqz@sdu.edu.cn

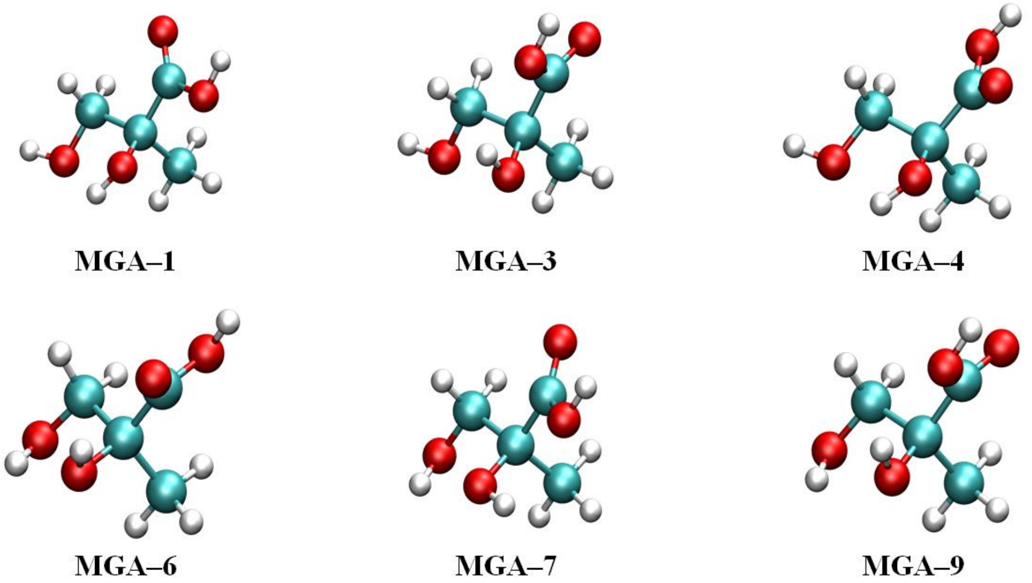

Fig. S1. The six configurations of MGA molecule.

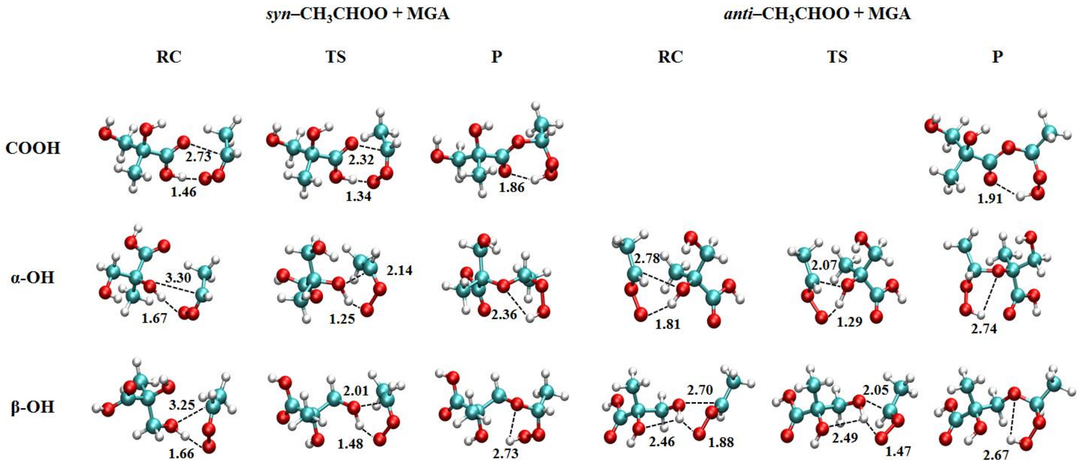

Fig. S2. The structures of bimolecular reaction complexes (RC), transition states (TS) and products (P).

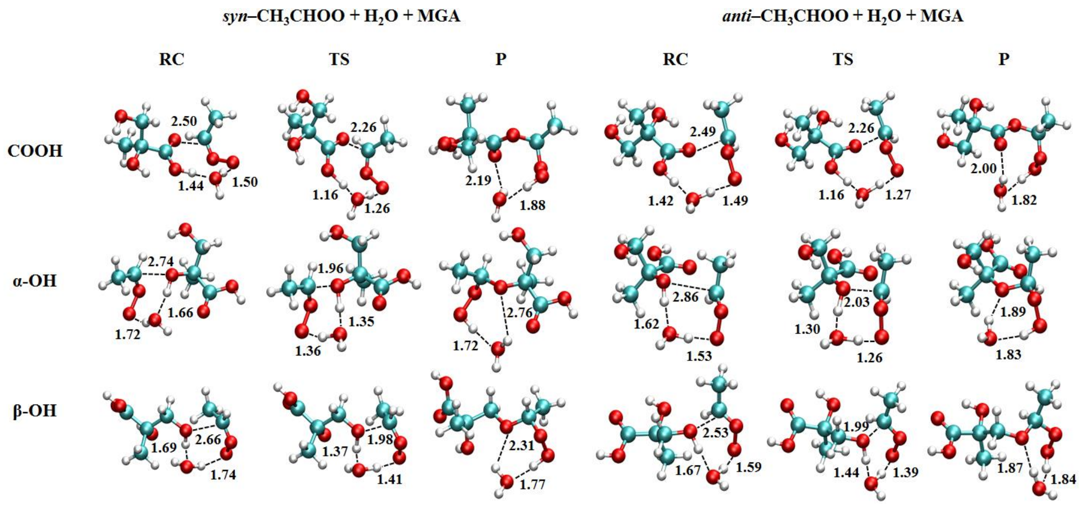

Fig. S3. The structures of water-mediated reaction complexes (RC), transition states (TS) and products (P).

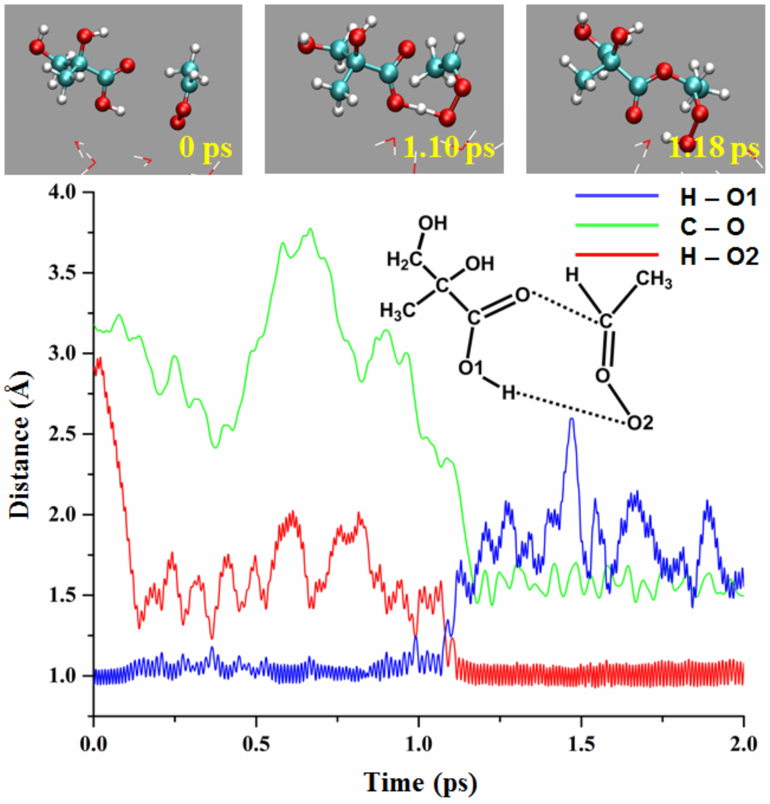

Fig. S4. The key bond length variations and snapshots of the gas-liquid interfacial reaction of *syn*-CH_3CHOO with the MGA COOH group.

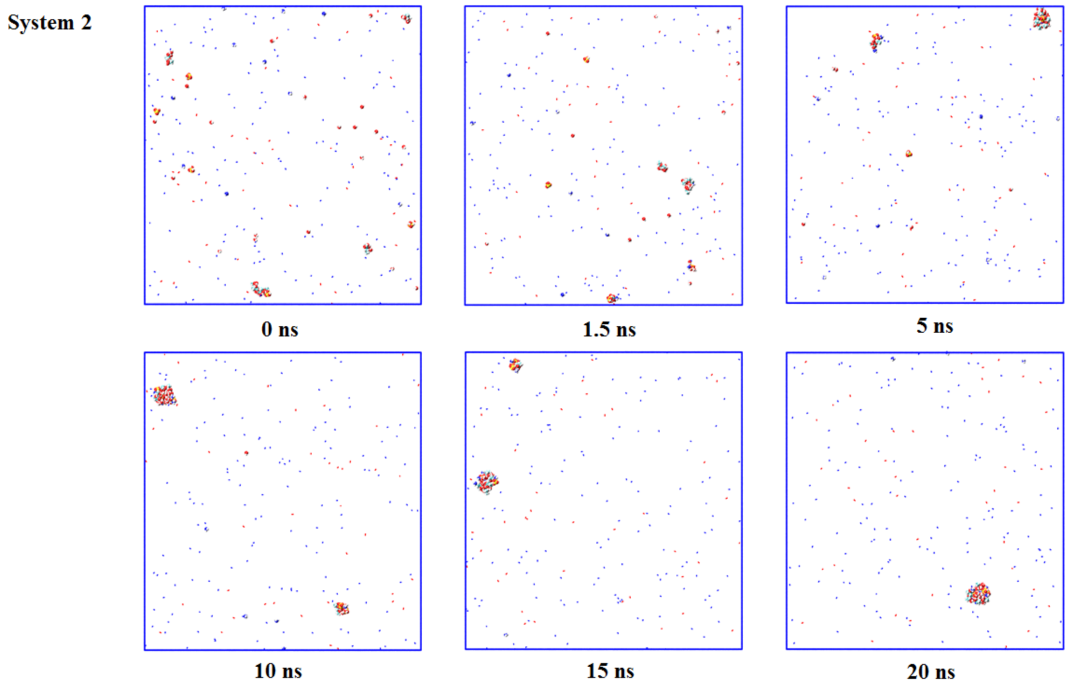

Fig. S5. The snapshots of nucleation simulation for the reaction product of *anti*-CH_3CHOO + MGA-β-OH.

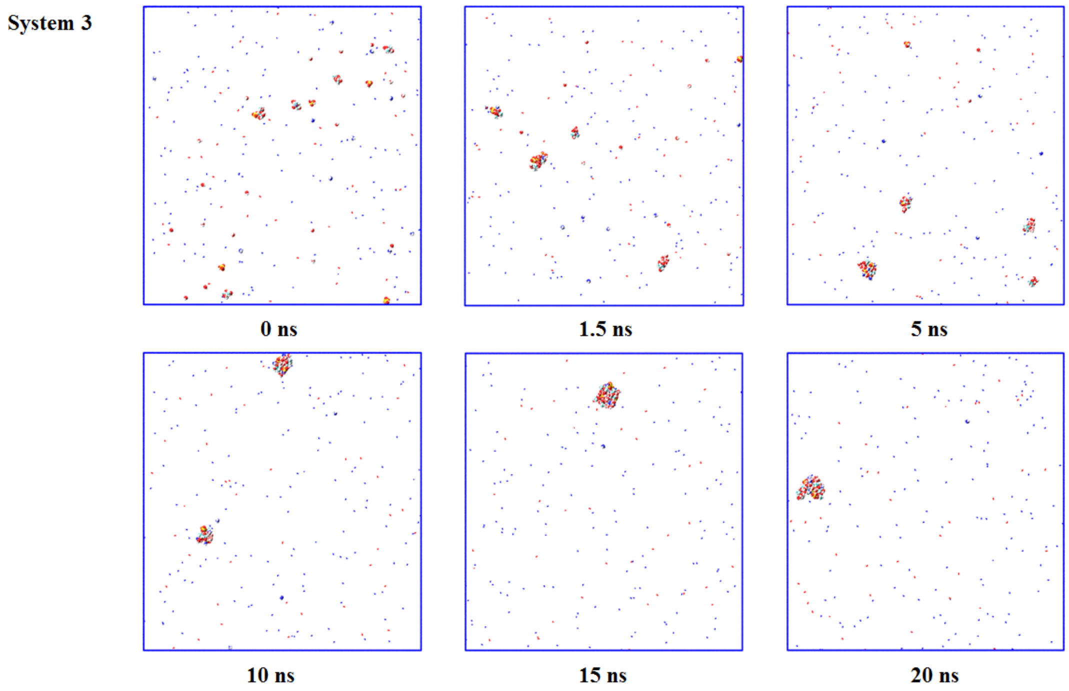

Fig. S6. The snapshots of nucleation simulation for the reaction product of *anti*-CH_3CHOO + MGA-α-OH.

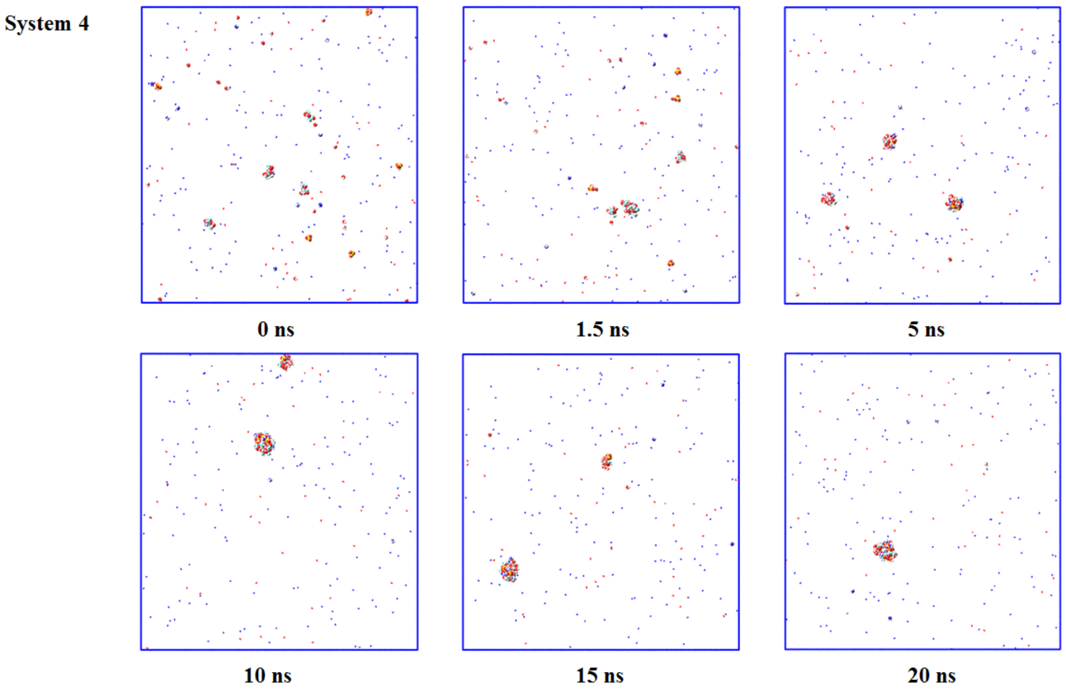

Fig. S7. The snapshots of nucleation simulation for the reaction product of *syn*-CH_3CHOO + MGA-COOH.

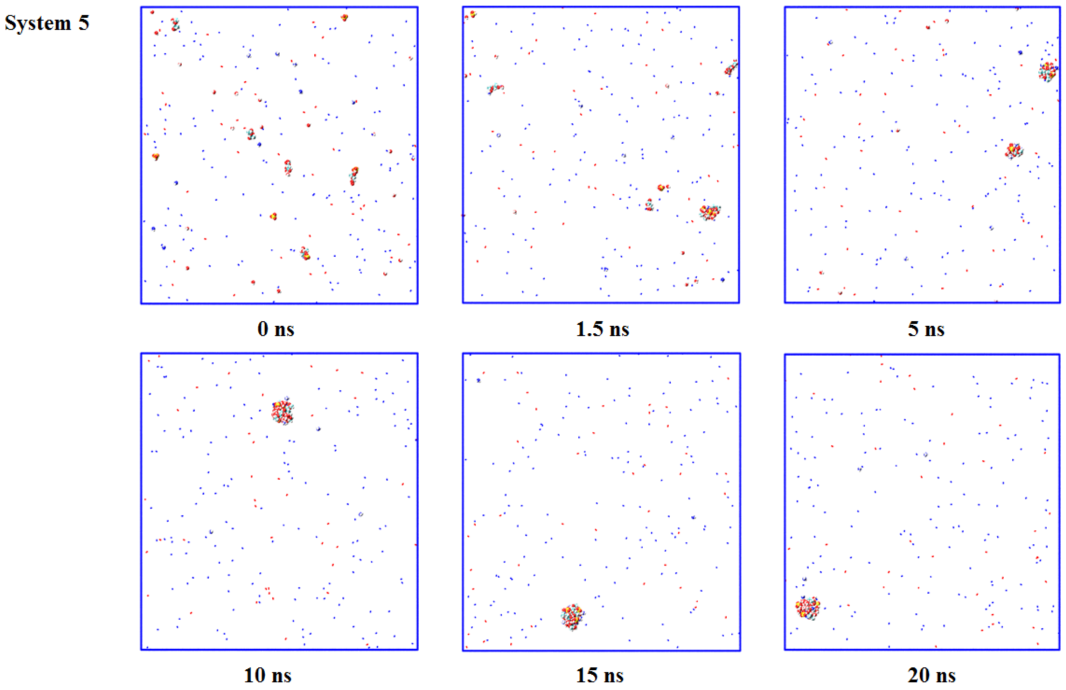

Fig. S8. The snapshots of nucleation simulation for the reaction product of *syn*-CH_3CHOO + MGA-β-OH.

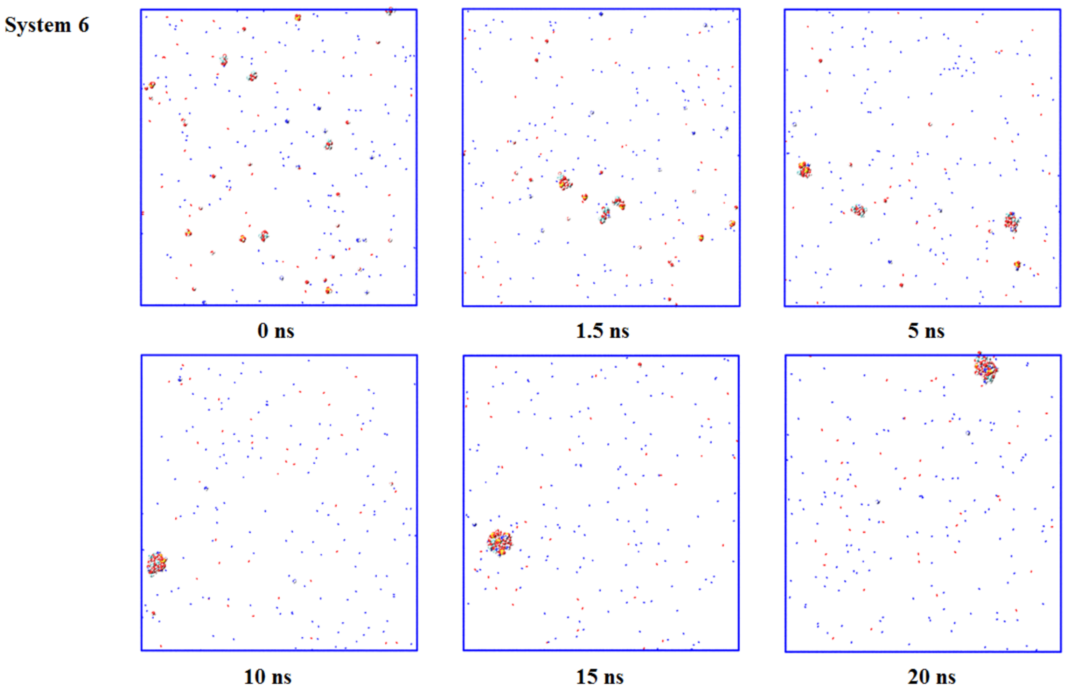

Fig. S9. The snapshots of nucleation simulation for the reaction product of *syn*-CH_3CHOO + MGA-α-OH.
